# Case Report: Anastrozole as a monotherapy for pre-pubertal children with non-classic congenital adrenal hyperplasia

**DOI:** 10.3389/fendo.2023.1101843

**Published:** 2023-03-02

**Authors:** Sandy C. Liu, Malavika Suresh, Mutaz Jaber, Yesica Mercado Munoz, Kyriakie Sarafoglou

**Affiliations:** ^1^ Department of Pediatrics, Division of Pediatric Endocrinology, University of Minnesota Medical School, Minneapolis, MN, United States; ^2^ Department of Experimental and Clinical Pharmacology, University of Minnesota College of Pharmacy, Minneapolis, MN, United States

**Keywords:** congenital adrenal hyperplasia, anastrozole, aromatase inhibitor, hydrocortisone, bone maturation, growth, androgen, estrogen

## Abstract

Most children with non-classic congenital adrenal hyperplasia (NC-CAH) due to 21-hydroxylase deficiency are asymptomatic and do not require cortisol replacement therapy unless they develop symptoms of hyperandrogenemia. The current practice is to treat symptomatic children with hydrocortisone aimed at suppressing excess adrenal androgen production irrespective of the child’s level of endogenous cortisol production. Once on hydrocortisone therapy, even children with normal cortisol production require stress dosing. Some children with NC-CAH may present with premature adrenarche, growth acceleration, and advanced bone age, but with no signs of genital virilization and normal endogenous cortisol production. In these cases, an alternative therapy to hydrocortisone treatment that does not impact the hypothalamic–pituitary–adrenal axis, but targets increased estrogen production and its effects on bone maturation, could be considered. Aromatase inhibitors (AIs), which block the aromatization of androgen to estrogen, have been used off-label in men with short stature to delay bone maturation and as an adjunct therapy in children with classic CAH. The use of AI as a monotherapy for children with NC-CAH has never been reported. We present three pre-pubertal female children with a diagnosis of NC-CAH treated with anastrozole monotherapy after presenting with advanced bone age, early adrenarche, no signs of genital virilization, and normal peak cortisol in response to ACTH stimulation testing. Bone age z-scores normalized, and all three reached or exceeded their target heights. Monotherapy with anastrozole can be an effective alternative in slowing down bone maturation and improving height outcomes in children with NC-CAH and normal adrenal cortisol production.

## 1 Introduction

Congenital adrenal hyperplasia due to 21-hydroxylase deficiency (21-OHD) is characterized by disruption in the pathway involved in cortisol and aldosterone production. Accumulated precursors above the enzymatic block are shunted to the androgen synthesis pathway resulting in excess androgen and, through aromatization, increased adrenal estrogen production. Conventionally, two distinct phenotypic entities of 21-OHD, the classic (severe) and the non-classic (NC-CAH; mild), have been described, although genotype and clinical phenotype correlations suggest that 21-OHD is a continuum of phenotypes and there is not always a clear phenotypic distinction (1). NC-CAH is associated with 30%–50% residual enzyme activity and usually present with androgen excess and either normal or suboptimal cortisol production (2). The majority of individuals with NC-CAH are asymptomatic. When symptomatic, children present with premature adrenarche, growth acceleration, and rapid bone maturation. If not treated, it can lead to early epiphyseal closure and short stature due to increased estrogen exposure, through aromatization of the elevated adrenal androgen. Post-natal virilization such as clitoral enlargement and penile enlargement can develop in NC-CAH patients who carry the P230L non-classic *CYP21A2* allele, as it is associated with more pronounced androgen excess (3). Children with NC-CAH are treated only if they are symptomatic. The current practice is to treat symptomatic patients with glucocorticoid supplementation aimed at suppressing excess adrenal androgen production irrespective of the child’s level of endogenous cortisol production (2).

Glucocorticoid therapy involves a number of logistic challenges especially in growing children. Treatment with hydrocortisone requires close monitoring and frequent dose adjustments to ensure adequate suppression and to minimize adverse sequelae of chronic glucocorticoid therapy on growth, weight, and blood pressure control (4–7). Furthermore, and importantly, chronic supplementation with glucocorticoids suppresses endogenous production of cortisol, resulting in patients requiring stress dosing during periods of illness, stress, or hospitalization.

Some children with NC-CAH may present with growth acceleration and advanced bone age but with no signs of genital virilization and normal cortisol production. In these patients, an alternative therapy to hydrocortisone treatment that does not impact the hypothalamic–pituitary–adrenal (HPA) axis, but targets increased estrogen production and its effects on bone maturation, could be considered. Aromatase inhibitors (AIs), which block the aromatization of androgen to estrogen, were first introduced as a treatment for estrogen-receptor-positive breast cancer but have been used off-label in children for treatment of short stature. Third-generation AIs such as anastrozole and letrozole have been reported to be effective for treatment of males with growth hormone deficiency (8), idiopathic short stature (9, 10), and/or constitutional delay of puberty (11, 12), and of children with disorders of puberty and advanced bone age (13, 14).

The use of AIs in children with 21-OHD has mostly been limited to adjunct therapy with hydrocortisone therapy (15, 16). Until now, there have not been any published studies or case reports on patients with NC-CAH treated with AI monotherapy. We report on growth, bone maturation, and pubertal progression of three pre-pubertal patients with symptomatic NC-CAH, but normal adrenal cortisol production, who were treated with anastrozole monotherapy instead of hydrocortisone therapy either alone or combined with AIs.

## 2 Case presentations

### 2.1 Case 1

Case 1 is a Caucasian female patient who was born prematurely at 24 weeks, with a post-natal course complicated by bronchopulmonary dysplasia, intraventricular hemorrhage, necrotizing enterocolitis, retinopathy of prematurity, patent ductus arteriosus ligation, oral aversion and reflux requiring gastrostomy tube and Nissen fundoplication, and right hemiplegic cerebral palsy. She presented to our multidisciplinary CAH clinic at age 3.5 years for the evaluation of axillary hair growth that began at approximately age 2, followed by adrenarche and development of body odor. On examination, she presented with normal female external genitalia, Tanner stage I breast development with coarse areolar hair, dark axillary hair, and Tanner stage III pubic hair. Her baseline morning endocrine workup showed elevated testosterone, 17-hydroxyprogesterone (17-OHP), androstenedione (D4A), dehydroepiandrosterone sulfate (DHEA-S), and pre-pubertal estradiol (E_2_) (**Table 1**). She had an advanced bone age of 6.8 years at a chronological age of 3.4 years (bone age z-score, 6.4). Her height z-score corrected for bone age was −4.06. On high-dose ACTH stimulation test, she had a normal cortisol response and elevated 17OHP (**Table 1**) consistent with NC-CAH. *CYP21A2* molecular testing showed that she was homozygous for the non-classic V281L (c.844G>T) pathogenic variant, and parental testing showed that both parents were carriers of this variant.

**Table 1:** Bone age z-scores, height z-scores, and hormonal levels at the initiation and end of anastrozole therapy.

Start of anastrozole therapy														End of anastrozole therapy					
									*ACTH stimulation test*										
	Age (yrs)	Bone age (years)	Bone age z-score	Corrected height z-score	17OHP <91 ng/dl	D4A ng/dL	T <2.5-10 ng/dl	DHEA-S <10-17 ug/dl	Estradiol 0-10 pg/ml	Baseline cortisol ug/dl	Stim. cortisol ug/dl	Baseline 17OHP ng/dl	Stim. 17OHP ng/dl	Age (yrs)	Bone age (yrs)	Bone age z-score	Corrected height z-score	FAH z-score	Target height z-score
Case 1	3.7	6.8	6.4	-4.33	307	230^a^	32	114	9	10	18	480	5340	14.5	14	-0.35	-0.63	-0.61	0.2
Case 2	3.9	6.3	4.0	-2.70	606	30^b^	12	28	7	8.5	19	180	6300	12.6	15	1.35	0.27	0.24	0.2
Case 3	6.7	8.8	2.9	-0.11	193	25^c^	7.3	193	9	7.7	19.3	193	9210	12.0	12	-0.23	2.19	2.30	2.0

All bone age X-rays were read using the Greulich and Pyle method by a pediatric radiologist and reviewed by KS. Bone age Z‐scores for each child were calculated using the mean skeletal age and standard deviation from Gruelich and Pyle. ACTH, adrenocorticotropic hormone; 17OHP, 17-hydroxyprogesterone; D4A, androstenedione; T, testosterone; DHEA-S, dehydroepiandrosterone sulfate; FAH, final adult height.

a Normal range, 80–190 ng/dl for Tanner stage III pubic hair.

b normal range <10–17 ng/dl for Tanner Stage I pubic hair.

c normal range 17-153 ng/dl for Tanner Stage II public hair.

Due to her prematurity and related comorbidities, she had a number of foreseen upcoming procedures and hospitalizations. The parents’ primary concern was that although treatment with cortisol would address her advanced bone maturation and growth acceleration, it would also require frequent stress dosing during periods of illness and for procedures due to secondary (iatrogenic) adrenal insufficiency. The parents elected to proceed with a trial of anastrozole monotherapy, since she had mild symptoms of androgen excess without any clinical or hormonal evidence of adrenal insufficiency.

**Figure 1:**
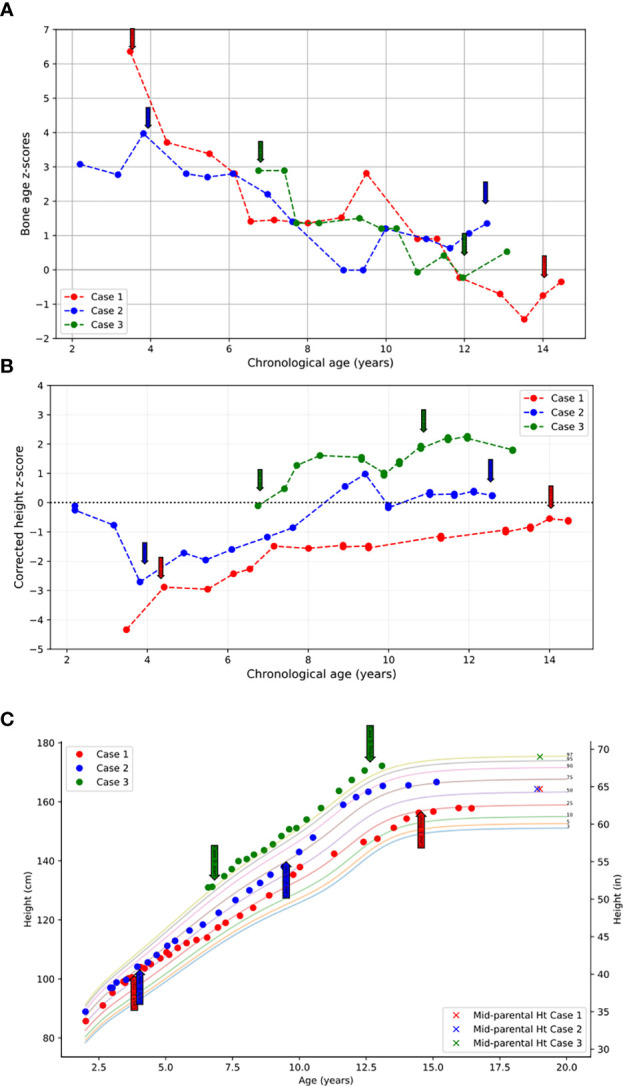
**(A)** Bone age z-scores plotted against chronological age throughout the course of treatment. **(B)** Height z-scores corrected for bone age plotted against chronological age throughout the course of treatment. **(C)** Heights plotted throughout course of treatment over the standardized pediatric growth chart as published by the CDC. Initiation and termination of anastrozole treatment is indicated with arrows.

**Figure 2:**
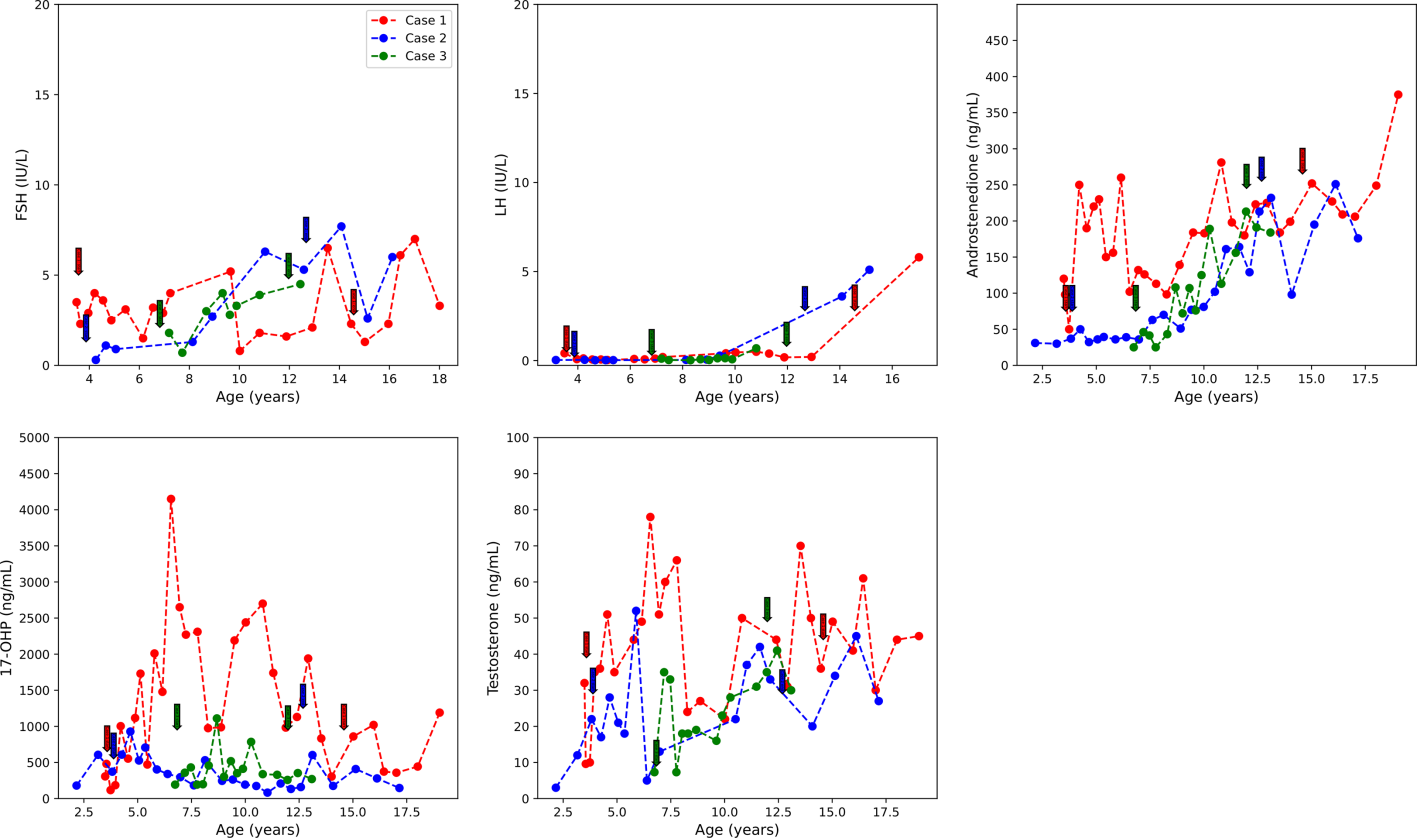
FSH, LH, D4A, 17-OHP, and testosterone concentrations over the course of treatment. Initiation and termination of treatment are indicated with colored arrows. D4A, androstenedione; 17-OHP, 17-hydroxyprogesterone. Initiation and termination of anastrozole treatment is indicated with arrows.

She was treated with anastrozole (1 mg daily) from age 3.7 years to age 14.5 years. Ongoing monitoring during her treatment course included liver function tests and bone age X-rays every 6 months, dual-energy X-ray absorptiometry (DXA scans), and hormonal monitoring including luteinizing hormone (LH), follicle-stimulating hormone (FSH), 17-OHP, D4A, and testosterone concentrations. During treatment, her liver transaminases were within normal range. Her bone age X-rays showed a slowing progression of bone maturation, resulting in a rapid correction in bone age z-scores (**Table 1**; **Figure 1A**). Serial DXA scans throughout and after treatment showed normal bone mineral density (**Table 2**). During this period (3.7–14.5 years), she was also noted to have age-appropriate linear growth (**Figures 1B, C**). Her LH and FSH remained within the expected range for her Tanner stage (**Figure 2**). She did report mild acne but otherwise tolerated anastrozole treatment without any adverse effects. During the course of treatment, she underwent nine surgical procedures requiring anesthesia. She did not receive stress dose glucocorticoids and did not have any complications from any of these procedures and surgeries.

**Table 2:** Serial bone mineral density and ovarian ultrasound measures.

Bone Mineral Density by DXA									Ovarian Ultrasound							
	Age (years)	BMD z-score	Age (years)	BMD z-score	Age (years)	BMD z-score	Age (years)	BMD z-score	Age (years)	Ovarian U/S	Age (years)	Ovarian U/S	Age (years)	Ovarian U/S	Age (yrs)	Ovarian U/S
Case 1	9.9	L1-L4: 0.4 TBLH: -1.1	12.5	L1-L4: -1.1 TBLH: -1.9	22.1	L1-L4: 0.6 R/L femur: -1.1 R/L hip: -0.3/-0.7	–	–	5.7	R 0.3 ml L 0.3 ml	10.5	R 6 ml L 3.2 ml	14.7	R 5.6 ml L 8.2 ml	18.9	R 8.7 ml L 7.2 ml
Case 2	4.9	L1-L4: n/d TBLH: -1.4	8	L1-L4: -0.6 TBLH: -0.8	11	L1-L4: -0.9 TBLH: -0.7	13.9	L1-L4: 0.1 TBLH: 0.0	3.9	R n/v L 0.5 ml	12	R 8 ml L 2.5 ml	16	R 11.3 ml L hem cyst*	17	R 10.3 ml L 13 ml
Case 3	9.9	L1-L4: 0.8 TBLH: 0.9	11	L1-L4: 0.5 TBLH: 1.0	13.1	L1-L4: 1.9 TBLH: 1.7	19.9	L1-L4: 1.2 TBLH: 1.8	6.7	R 1.4 L 1.2	10.7	R 1.8 L 2.5	13.4	R 9.1 L 6.7	–	–

We defined normal BMD as having a BMD Z-score in the normal range (between +2 and −2 SDS). DXA, dual-energy X-ray absorptiometry; R, right; L, left; L1-L4, lumbar vertebrae; TBLH, total body less head.

*5.2 cm hemorrhagic cyst. "-" means "n/a".

At age 9.8 years, she was noted to have acne and findings consistent with onset of central puberty including breast bud development, an LH level of 0.41 mIU/ml, and increased ovarian volumes in pelvic ultrasound. At age 9.9 years, she was started on a GnRH analog, histrelin acetate, to block pubertal progression due to concerns about her age and her emotional immaturity. At age 13, GnRH receptor analog therapy was stopped and puberty progressed. An ovarian ultrasound at age 13.8 years was normal (**Table 2**), and she underwent menarche 2 months later.

At the time of stopping anastrozole (age 14.5 years), her bone age z-score was −0.35, a significant improvement from when she began anastrozole at age 3.7 years with a bone age z-score of 6.4 (**Table 1**). At 18 years and 11 months, she was 158 cm tall (height z-score of −0.68), which was close to her target height of 164.4 (target height z-score of 0.2) (**Table 1**) (**Figures 1B, C**).

### 2.2 Case 2

Case 2, the younger sibling of Case 1, was diagnosed with NC-CAH at age 2 after undergoing *CYP21A2* molecular testing for the familial pathogenic variants. She was found to be homozygous for the V281L (c.844G>T) pathogenic variant, the same as her sibling. She presented to the endocrinology clinic at 3 years of age with a report of vaginal discharge. Workup at that time revealed an advanced bone age of 4.2 years at a chronological age of 3.1 years (bone age z-score of 2.8). She had no signs of puberty or adrenarche (Tanner stage I for breast and pubic hair development).

Follow-up evaluation at 3.9 years of age revealed an advanced bone age of 6.3 years (bone age z-score of 4.0). Laboratory evaluation revealed an elevated testosterone, 17-OHP, D4A, normal DHEAS, and pre-pubertal E_2_ (**Table 1**). On high-dose ACTH stimulation test, she had a normal cortisol response and elevated 17-OHP consistent with NC-CAH (**Table 1**). Physical examination showed no signs of pubertal progression—Tanner stage 1 breasts and pubic hair. Since her bone age continued to advance, she was started on anastrozole monotherapy. For similar reasons as her older sibling, the parents opted not to treat her with hydrocortisone.

Following the initiation of anastrozole (1 mg daily), her bone age z-scores (**Table 1** and **Figure 1A**) and her height gradually normalized (**Figures 1B, C**). Throughout the treatment, her FSH and LH concentrations and liver transaminases remained within the expected range for her Tanner stage of pubertal development (**Figure 2**). Her 17-OHP, D4A, and testosterone levels remained stably elevated throughout the therapy without a significant increase correlating with initiation of anastrozole (**Figure 2**).

At age 7, she reported facial acne and adrenarche as evidenced by Tanner stage II pubic hair development. At age 9.4 years, she had onset of puberty based on Tanner stage II breast development. She underwent menarche at a chronological age of 11.8 years.

After stopping anastrozole (age 12.6 years), her bone age was 15 years with a z-score of 1.35 compared to when she began anastrozole at age 3.9 years with a bone age z-score of 4.0. Her height was 163.4 cm. At age 18, her final adult height was 167.5 (height z-score, 0.7), which exceeded her mid-parental height of 164.4 cm (target height z-score, 0.2) (**Table 1**) (**Figures 1B, C**). Her bone density by DXA and her ovarian ultrasound remained normal at the end of anastrozole therapy (**Table 2**).

### 2.3 Case 3

Case 3, a 6.7-year-old healthy Caucasian female patient, presented to her primary care physician (PCP) clinic for evaluation of 6 months of greasy hair, open comedones over the nose, mood swings, increased body odor, and some sparse pubic hair without breast enlargement. Workup by her PCP revealed a bone age of 8.8 years at chronological age of 6.7 years (z-score of 3.01) and elevated 17-OHP at 680 ng/dl (normal range, <91 ng/dl). Physical examination revealed Tanner stage II pubic hair without other signs of virilization. Family history was positive for a female cousin with facial hair, short stature, and obesity, and an aunt with facial hair and obesity. Due to concern for NC-CAH, she was referred to our CAH clinic. Laboratory evaluation revealed prepubertal E_2_ and testosterone levels and elevated 17-OHP, D4A, and DHEAS. On high-dose ACTH stimulation test, she had a normal cortisol response and elevated 17-OHP consistent with NC-CAH (**Table 1**). *CYP21A2* molecular testing showed compound heterozygosity with a V281L (c.844G>T) pathogenic variant and a 30-kb deletion. Parental testing showed that her mother was a carrier of the 30-kb deletion and her father a carrier of the V281L (c.844G>T) pathogenic variant.

Treatment options were discussed, and the parents elected to proceed with anastrozole monotherapy given their child’s normal cortisol levels. Anastrozole (1 mg daily) was initiated at age 6.8 years. She had a baseline pelvic ultrasound showing normal pre-pubertal ovaries (**Table 2**). During the course of treatment, she did develop facial acne necessitating treatment with tretinoin at age 8 but reported no other signs of androgen excess. Ongoing monitoring during treatment included adrenal steroid levels and liver transaminases, both of which stable throughout the course of treatment. (**Figure 2**). Overall, her bone ages, which were monitored every 6-12 months, gradually normalized following the initiation of anastrozole treatment and showed little interval maturation thereafter. (**Table 1**) (**Figure 1A**). Pubertal onset occurred at 9.3 years. Her bone density by DXA and her ovarian ultrasound remained normal at the end of anastrozole therapy (**Table 2**).

At age 12 years, anastrozole was stopped with a bone age of 12 years (bone age z-score of −0.23) and a height of 167.5 cm. She had menarche at 12.3 years. Eventually, she achieved an adult height of 180.3 cm (height z-score, 2.3) compared to a mid-parental height of 176.5 cm (target height z-score, 2.0) (**Table 1**) (**Figures 1B,C**).

## 3 Discussion

The negative impact of supraphysiological glucocorticoid supplementation in children with NC-CAH in order to control increased adrenal sex production is described in a recent report by Wasniewska et al., where untreated children presented a trend (p = 0.053) toward higher adult height z-scores compared to those who were on hydrocortisone replacement (17). We present a novel approach for the treatment of three pre-pubertal female children diagnosed with NC-CAH with early adrenarche and advanced bone age but intact cortisol production. Instead of hydrocortisone therapy, either alone or combined with an AI, we used anastrozole as a monotherapy, as it does not impact the HPA axis and instead targets increased estrogen production and its effects on bone maturation and growth.

The three patients discussed in this report are the only patients with NCCAH who presented to our center with premature adrenarche, growth acceleration, and advanced bone age but with no signs of genital virilization and normal endogenous cortisol production after high-dose ACTH stimulation. As such, they are the only patients that have ever been offered anastrozole monotherapy as an alternative treatment to hydrocortisone.

After initiation of treatment with anastrozole, bone maturation slowed in all three children leading to bone ages more closely aligned with their chronologic ages at the end of AI monotherapy. Their average bone age z-score at the initiation of therapy was 4.43 and decreased to 0.77 at the time of stopping AI treatment. All three female children achieved adult heights comparable to their genetic potential.

Children with classic CAH and symptomatic NC-CAH are at risk of chronically elevated estrogen exposure through aromatization of adrenal androgen due to shortcomings of current therapy in children. Hydrocortisone, the recommended glucocorticoid for treatment of children with CAH, has a short half-life leading to alternating periods of hyper- and hypocortisolemia and resultant increased androgen and estrogen exposure throughout the day (18, 19). Chronic increased estrogen exposure can result in rapid bone maturation, early epiphyseal closure, and decreased final adult height, as ultimate fusion of the growth plate is estrogen dependent. Halper et al. reported that children with CAH treated with hydrocortisone and anastrozole had significant slowing of the rate of bone age maturation as evidenced by the change of bone age z-scores from 4.3 to 1.9 on anastrozole (15).

The use of aromatase inhibitors in children has the potential to decrease bone mineral density, as estrogen has a significant role in bone development and turnover through stimulation of osteoblast activity and survival and inhibition of osteoclast activity and differentiation (20). Serial DXA monitoring in our three cases showed normal bone density throughout the study and after discontinuation (**Table 2**) similar to other non-CAH studies that used AIs in children (9–11, 14). Halper et a.l did not find significant changes in bone mineral density in 56 children with CAH treated for an average of 5.2 [SD, 2.2] years with anastrozole and hydrocortisone when compared to children with CAH who were treated just with hydrocortisone (15). In addition, both groups had normal bone density when compared with lunar DXA reference data. The authors of the study speculated that increased androgen bone exposure due to inhibition of its aromatization to estrogen in children with CAH treated with anastrozole may compensate for the effect of decreased estrogen levels on bone.

Current evidence suggests that both androgen and estrogen may have a positive effect on the bone, as they both can stimulate osteoblastic activity, counteract osteoblast apoptosis, and stimulate osteoclast apoptosis (20, 21). Androgen action on the bone can be mediated directly *via* binding at the androgen receptors (ARs) and indirectly *via* binding to estrogen receptors (ERα and ERβ) after aromatization to estrogen in adipose or other peripheral tissues with aromatase enzyme activity (22, 23).

The HPG axis is normally dormant during childhood, with HPG activation and subsequent pulsatile GnRH release and LH/FSH release by the pituitary gland leading to puberty onset. Given that estrogen is involved in the negative feedback of the HPG axis in those who have undergone central puberty, treatment with an AI could potentially lead to elevated GnRH, LH, and FSH, overstimulation of the ovaries and cyst formation, and even a change in the timing of puberty onset. All three patients underwent serial ovarian ultrasounds to screen for ovarian hyperplasia during and at the end of anastrozole therapy (**Table 2**). None of the patients had abnormally enlarged ovarian volumes or changes in ovarian morphology except for case 2 who had a finding of a hemorrhagic ovarian cyst, which self-resolved. Patients with CAH are at increased risk for PCOS even after postnatal therapy with hydrocortisone most likely due to the effect on fetal programming of elevated adrenal androgen *in utero* (24) and the impact of chronically elevated androgen between hydrocortisone dosing as a result of hydrocortisone’s short half-life. To the extent that monotherapy with anastrozole may further increase the risk of PCOS needs to be examined.

Studies investigating AI treatment in non-CAH male youth showed that AI treatment did not affect the onset of puberty in pre-pubertal youth (9, 11). There is scarce information on the effect of AI treatment on pre-pubertal female children. In our cohort of NC-CAH patients, LH, FSH were regularly measured, and none of the three showed significant gonadotropin elevation with initiation of anastrozole therapy or had early onset puberty. In fact, Case 1, although she was at increased risk for precocious puberty due to her history of cerebral palsy (25) and prematurity, had normal timing of puberty onset at age 9.8 years. Estradiol levels were not measured serially because of the reported substantial variability in E_2_ especially at these low E_2_ concentrations (26). In our three patients, baseline E_2_ levels were prepubertal even in the presence of advanced bone maturation, suggesting that serum E_2_ may not be the most reliable measure of the effect of estrogen on bone.

While the strength of the data is limited by the small number of subjects, the three cases suggest that anastrozole as a monotherapy can be a viable alternative to hydrocortisone in children with NC-CAH and normal cortisol production. Anastrozole monotherapy addresses rapid bone age maturation and risk of early epiphyseal closure without affecting endogenous cortisol production, thereby eliminating the need for hydrocortisone stress dosing. Future longitudinal prospective studies examining growth and bone mineral density outcomes and PCOS risk of patients with NCCAH with normal endogenous cortisol and no genital virilization on AI monotherapy vs. hydrocortisone vs hydrocortisone and AI are needed.

## Data availability statement

The original contributions presented in the study are included in the article/supplementary material. Further inquiries can be directed to the corresponding author.

## Ethics statement

The studies involving human participants were reviewed and approved by University of Minnesota Institutional Review Board. The patients/participants provided their written informed consent to participate in this study. Written informed consent was obtained from the participant/patient(s) for the publication of this case report.

## Author contributions

All authors contributed to the conception and design of the report. Data collection and analysis were performed by SL, MS, MJ, YM, and KS. The first draft of the manuscript was written by SL, and all authors commented on previous versions of the manuscript. All authors contributed to the article and approved the submitted version.
